# The stressosome, a caspase‐8‐activating signalling complex assembled in response to cell stress in an ATG5‐mediated manner

**DOI:** 10.1111/jcmm.16840

**Published:** 2021-08-07

**Authors:** Katarzyna Mnich, Izabela Koryga, Karolina Pakos‐Zebrucka, Melissa Thomas, Susan E. Logue, Leif A. Eriksson, Adrienne M. Gorman, Afshin Samali

**Affiliations:** ^1^ Apoptosis Research Centre NUI Galway Galway Ireland; ^2^ School of Natural Sciences NUI Galway Galway Ireland; ^3^ CÚRAM SFI Research Centre for Medical Devices NUI Galway Galway Ireland; ^4^ Department of Chemistry and Molecular Biology University of Gothenburg Göteborg Sweden; ^5^ Department of Human Anatomy and Cell Science Rady Faculty of Health Sciences Max Rady College of Medicine University of Manitoba Winnipeg MB Canada; ^6^ Research Institute in Oncology and Hematology Cancer Care Manitoba Winnipeg MB Canada

**Keywords:** apoptosis, autophagy, caspase, cell stress, integrated stress response

## Abstract

Stress‐induced apoptosis is mediated primarily through the intrinsic pathway that involves caspase‐9. We previously reported that in caspase‐9‐deficient cells, a protein complex containing ATG5 and Fas‐associated death domain (FADD) facilitated caspase‐8 activation and cell death in response to endoplasmic reticulum (ER) stress. Here, we investigated whether this complex could be activated by other forms of cell stress. We show that diverse stress stimuli, including etoposide, brefeldin A and paclitaxel, as well as heat stress and gamma‐irradiation, caused formation of a complex containing ATG5‐ATG12, FADD and caspase‐8 leading to activation of downstream caspases in caspase‐9‐deficient cells. We termed this complex the ‘stressosome’. However, in these cells, only ER stress and heat shock led to stressosome‐dependent cell death. Using *in silico* molecular modelling, we propose the structure of the stressosome complex, with FADD acting as an adaptor protein, interacting with pro‐caspase‐8 through their respective death effector domains (DEDs) and interacting with ATG5‐ATG12 through its death domain (DD). This suggests that the complex could be regulated by cellular FADD‐like interleukin‐1β‐converting enzyme–inhibitory protein (cFLIP_L_), which was confirmed experimentally. This study provides strong evidence for an alternative mechanism of caspase‐8 activation involving the stressosome complex.

## 1 INTRODUCTION

Caspase‐8 is a cysteine protease that was first identified as the apical caspase in the induction of extrinsic apoptosis.^1^ Since then, a role for caspase‐8 has emerged in diverse cellular processes in cancer biology and anticancer therapies, such as necroptosis,^2^ autophagy‐dependent cell death,^3^ inflammation and cytokine release,^4^ angiogenesis,^5^ cell adhesion and migration^6^ and immune cell homeostasis.^7^ Thus, caspase‐8 is important for tumour development, progression, therapy resistance and the development of the tumour microenvironment.

Pro‐caspase‐8 comprises a pro‐domain at the N‐terminus containing tandem death effector domains (DED1 and DED2), connected to a long domain and a short domain at the C‐terminus.^8^ The canonical mechanism of caspase‐8 activation during initiation of extrinsic (or death receptor‐mediated) apoptosis is through ligand‐induced oligomerization of death receptors (DR) such as Fas or tumour necrosis factor–related apoptosis‐inducing ligand receptors.^8^ This leads to recruitment of Fas‐associated death domain (FADD), which forms homotypic interactions with the DRs through their respective death domains (DDs) and with pro‐caspase‐8 through their respective DEDs. Together these form the death‐inducing signalling complex where caspase‐8 undergoes proximity‐induced auto‐activation.^8^

Several non‐canonical or alternative platforms for activation of caspase‐8 have been identified, many of which demonstrate a requirement for FADD as an adaptor protein.^3^, ^9^, ^10^, ^11^ Several studies describe an involvement of components of the autophagy pathway such as p62^3^ or ATG5,^3^, ^9^, ^10^ while some report a role for autophagosomal membranes.^3^, ^12^, ^13^ Furthermore, unmitigated endoplasmic reticulum (ER) stress has been shown to activate caspase‐8 by inducing ligand‐independent intracellular DR5 activation, which is regulated by the integrated stress response (ISR) mediator, CHOP.^14^ We reported that caspase‐9 deficiency and blockade of the intrinsic apoptosis pathway can unmask an alternative caspase‐8‐dependent cell death pathway in response to ER stress. This caspase‐8 activation was independent of external DR ligation, but required interaction with FADD and ATG5 to form an intracellular caspase‐8‐activating complex.^10^

Here, we investigated whether ER stress is a unique inducer of the ATG5‐ATG12:FADD:pro‐caspase‐8 complex formation in caspase‐9‐deficient cells or whether it can assemble as a result of other cellular stresses. We demonstrate that in *Casp9* ^−/−^ cells, a range of stress stimuli can activate caspase‐8 through the formation of a ATG5‐ATG12:FADD:pro‐caspase‐8 complex, which we termed the stressosome. Using molecular modelling, we generated a model supporting the interaction of caspase‐8 with FADD and interaction of the DD region of FADD with ATG5‐ATG12. Caspase‐8 activation required ATG5 but was independent of autophagy. Although activation of the ISR was a common feature of the various stress stimuli, it did not mediate pro‐caspase‐8 activation. We further demonstrated that stressosome‐induced activation of caspase‐8 was inhibited by cellular FLICE‐inhibitory protein (cFLIP).

## 2 MATERIALS AND METHODS

### 2.1 Cell culture and treatments

Caspase‐9‐deficient mouse embryonic fibroblasts (*Casp9* ^−/−^ MEFs) and matched *Casp9* ^+/+^ MEFs were kindly gifted by Prof. Tak Mak (University of Toronto, Canada). Cells were cultured in high‐glucose Dulbecco's modified Eagle's medium (DMEM) (Sigma‐Aldrich, Saint Louis, Missouri, USA, D6429) supplemented with heat‐inactivated 10% (v/v) foetal bovine serum (FBS) (Sigma‐Aldrich, F7524), 100 U/ml penicillin, 100 mg/ml streptomycin (Sigma‐Aldrich, P0781), 2 mM L‐glutamine (Sigma‐Aldrich, G7513), non‐essential amino acids (Sigma‐Aldrich, M7145) and sodium pyruvate (Sigma‐Aldrich, S8636). Human embryonic kidney HEK 293T cells (ATCC, CRL‐11268) were cultured in DMEM supplemented with 10% FBS, 100 U/ml penicillin and 100 mg/ml streptomycin. All cells were maintained at 37°C, 5% CO_2_ in a humidified incubator.

To induce cellular stress, MEFs were seeded at the required density 24 h prior to treatment. The culture medium was then replaced with fresh medium containing 0.3 μg/ml brefeldin A (Sigma‐Aldrich, B6542), 50 μM etoposide (Sigma‐Aldrich, E1383) or 1 μM paclitaxel (Sigma‐Aldrich, T7402). Chloroquine (Sigma‐Aldrich, C6628) was used at 20 μM. ISRIB (Sigma‐Aldrich, SML0843) was added every 48 h at 200 nM. Spautin‐1 (Sigma‐Aldrich, SML0440) and rapamycin (LC Laboratories, Woburn, MA, USA, R‐500) were added every 24 h at 10 μM and 400 nM, respectively. For γ‐irradiation, cells were trypsinized and kept in suspension during γ‐irradiation (33 Gγ) with a caesium‐137 source (Mainance, Hampshire, UK). Cells were collected by centrifugation at 300 *× g* for 5 min and then seeded at the required density. For heat shock, cells were trypsinized and resuspended in DMEM into 50 ml tubes, which were immersed completely for 45 min in a preheated water bath with a circulating pump at 43.5 °C. Afterwards, they were pelleted at 300 *× g* for 10 min, resuspended in fresh DMEM and seeded at required densities.

### 2.2 Immunoblotting

Cells were lysed in whole cell lysis buffer (100 mM Tris‐HCl pH 6.8, 20% (v/v) glycerol, 4% (w/v) SDS, 0.1% (w/v) bromophenol blue, 2.5% (v/v) β‐mercaptoethanol). Proteins were separated by SDS‐PAGE and transferred to nitrocellulose membranes (GE Healthcare, Munich, Germany), which were then blocked in 5% (w/v) non‐fat milk dissolved in PBS containing 0.1% (v/v) Tween 20 (Sigma‐Aldrich, P5927). Membranes were probed with specific antibodies against: caspase‐9 (Cell Signaling Technology (CST), Danvers, MA, USA, #9508), FADD (Santa Cruz, Dallas, USA, sc‐6036), cleaved caspase‐8 (CST, #8592), caspase‐8 (CST, #4790), caspase‐3 (CST, #9662), Atg5 (CST, #12994), light‐chain 3 (LC3) (Sigma‐Aldrich, L7543), p‐eIF2α (CST, #3398), eIF2α (CST, #5324), ATF4 (CST, #9508), CHOP (CST, #2895), FLAG (Sigma, F1084) and actin (Sigma‐Aldrich, A2066). Horseradish peroxidase (HRP)–conjugated antibodies were from Jackson ImmunoResearch, Ely, UK: anti‐rabbit (111–035–003), anti‐mouse (115–035–003) and anti‐goat (705–035–003). The signal was visualized using enhanced chemiluminescence reagent (PerkinElmer, Waltham, Massachusetts, USA, NEL102001EA) or ImmunoCruz Luminol Reagent (Santa Cruz, sc‐2048).

### 2.3 Propidium iodide/ToPro3 staining

Membrane permeability was assessed using propidium iodide (PI) or ToPro3 staining. Briefly, cells were harvested by trypsinization and after 15 min of recovery at 37 °C the cells were resuspended in PBS containing 50 μg/ml PI (Sigma‐Aldrich, P4170) or 1 μM ToPro3 (Thermo Fisher Scientific, Dublin, Ireland T3605). Samples were analysed using a BD Accuri C6 flow cytometer (Becton Dickinson Biosciences) with C flow software V.1.0.264.15.

### 2.4 shRNA knockdown

Lentivirus for pLKO empty vector, shRNA vector against mouse *Casp8* (Sigma‐Aldrich, #TRCN0000012243), pGIPZ empty vector (Open Biosystems, RHS4349) and shRNA vector against mouse *Atg5* (Open Biosystems, RMM4431‐99342719) were generated by co‐transfecting plasmids with second‐generation lentivirus packaging system (Addgene, Watertown, Massachusetts, USA, pMD2.G, #12259; psPAX2, #12260; pRSV‐Rev, #12253) using JET PEI transfection reagent (Polyplus‐Transfection, Illkirch, France, #101‐01N) into HEK 293T cells. Virus‐containing supernatants were collected and filtered through 0.22 μm filter. MEFs were transduced in the presence of 5 μg/ml of polybrene (Merck Millipore, Cork, Ireland, TR‐1003‐G). Transduced cells were selected for 72 h in 5 μg/ml of puromycin (Sigma‐Aldrich, P8833) and in 2 μg/ml puromycin for subsequent two passages.

### 2.5 Immunoprecipitation

*Casp9*^−/−^ MEFs were seeded at the 3 x 10^4^ cells/cm^2^ density 24 h prior to the treatment. Boc‐D‐FMK at 20 μM (Cambridge Bioscience, Cambridge, UK, 1160–5) was added 24 h after stress induction to inhibit caspases. Cells were harvested 72 h after stress induction and lysed in 0.5 ml of ice‐cold NP‐40 lysis buffer (150 mM NaCl, 1% NP‐40, 10% glycerol, 10 mM Tris pH 8.0) supplemented with cOmplete™ Mini Protease Inhibitor Cocktail (Roche, Basel, Switzerland, 11836153001). The protein complex was immunoprecipitated using Dynabeads® M‐270 Epoxy (Invitrogen, Carlsbad, CA, USA, Kit number 143.21D) according to the manufacturer's instructions. Briefly, 1 mg of Dynabeads was conjugated to anti‐FADD (Santa Cruz, sc‐6036), anti‐ATG5 (CST, 8540) antibody, goat IgG control (Santa Cruz, sc‐2028) or rabbit IgG (Jackson ImmunoResearch, Ely, UK, 011‐000‐003) overnight at 4 °C. Dynabeads were washed in ice‐cold NP‐40 lysis buffer and combined with cell lysates on a rotor at 4 °C for 4 h. Following the incubation, the beads were washed and 50 μl of elution buffer was added. Beads were rotated at 4 °C for 5 min and precipitated on a magnet. The supernatants were transferred to fresh microfuge tubes and diluted in 50 μl of 2X sample buffer (4% (w/v) SDS, 120 mM Tris‐HCl, 10% (v/v) glycerol, 100 mM DTT and bromophenol blue).

### 2.6 siRNA transfection

ON‐TARGET plus SMART pool siRNA against mouse *Atf4* (Dharmacon, Lafayette, Colorado, USA, L‐042737‐01‐0005), *DDit3* (CHOP) (Dharmacon, L‐062068‐00‐0005) and a non‐targeting control (Dharmacon, D‐001810‐10‐20) were resuspended in 1X RNase buffer (60 mM KCl, 6 mM HEPES pH 7.5, 0.5 mM MgCl_2_), aliquoted and stored at −80 °C. MEFs were transfected with 20 nM of *Atf4* siRNA and 25 nM *DDit3* siRNA using DharmaFECT1 transfection reagent (Dharmacon, T‐2001‐01). The RNA‐DharmaFECT1 complexes were incubated at room temperature for 20 min. The complexes were added drop wise to the cells and incubated for 5 h at 37 °C. Media was changed, and 24 h later, cells were used for experiments.

### 2.7 cFLIP overexpression

The MEFs were transfected with a pME18S‐Flag vector containing a CDS for mouse cFLIP long isoform (kindly provided by Prof Kazuhiro Sakamaki, Kyoto University, Japan) using Lipofectamine 3000 transfection reagent (Thermo Fisher, #L3000008) at DNA‐to‐lipid ratio of 1:2. The media was removed 6 h after transfection. Transfected cells were allowed to recover for 24 h prior to any additional treatment. For heat shock treatment and γ‐irradiation exposure, cells were treated, seeded, let to recover for 4 h and followed with transfection with Lipofectamine 3000 which was performed as described above.

### 2.8 Protein‐protein docking

Human FADD Death Domain (PDB: 1E3Y) and human ATG5‐ATG12 complex (PDB: 4GDK) were prepared using Maestro Schrodinger (Schrödinger Release 2020‐4: Maestro, Schrödinger, LLC, New York, NY, 2020). Protein‐protein docking was performed using webservers PatchDock^15^ for protein‐protein docking and FireDock^16^ for flexible protein refinement and scoring of the obtained protein complexes. No constraints were applied on the proteins. The best poses were selected based on the lowest docking score. The HADDOCK protein‐protein docking webserver^17^ was used as a non‐blind docking approach to investigate FADD and caspase‐8 interaction. Amino acid described by Hughes and collaborators were in this case selected as constraints for the protein‐protein docking.^18^

### 2.9 Molecular dynamics simulation

Desmond molecular dynamic (MD) simulation engine^19^ was used to investigate how the FADD Death Domain behaved over the time in the ATG5‐ATG12 complex. The OPLS3e force field was used.^20^ Complex solvation was performed using TIP3P water models^21^ positioned in a cubic box extending 10 Å from nearest protein atom. Na^+^ and Cl^−^ ions were added to balance the system charge and to obtain a final physiological concentration of 150 mM of NaCl. The temperature and the pressure were kept constant at 300 K and 1 atmosphere, respectively. The simulations were run in triplicate for 100 ns under periodic boundary conditions.

### 2.10 Residue scanning

Residue hot spots were identified using Schrodinger‐BioLuminate residue scanning calculation.^22^ Mutated residues inducing the largest change in protein binding affinity (ΔAffinity, in kcal/mol) indicated that they have a significant contribution to the interaction between FADD and ATG5‐ATG12.

### 2.11 Statistical analysis

Images are representative of at least three independent experiments. Graphs show an average of at least three biological repeats. Error bars represent standard error of mean (SEM). Significance was determined using two‐way ANOVA, with *p* value <0.05 being considered significant and annotated by *.

## 3 RESULTS

### 3.1 Diverse inducers of cell stress activate caspase‐3 in *Casp9^−/−^* MEFs in a manner that is dependent on caspase‐8 and ATG5

**FIGURE 1:**
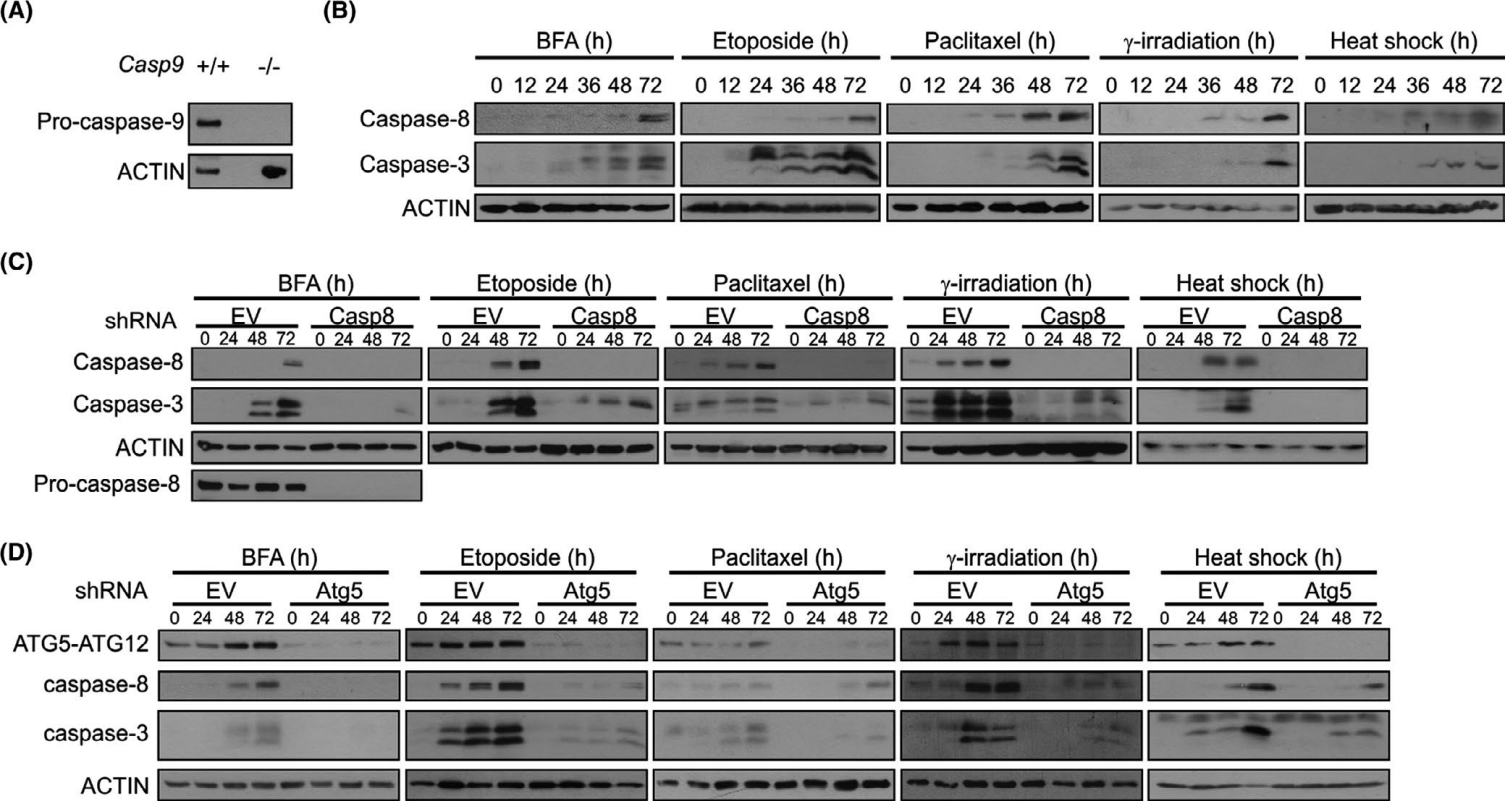
Caspase activation by different cell stressors in *Casp9* ^−/−^ MEFs and effect of knockdown of caspase‐8 or ATG5. (A) Whole cell lysates from *Casp9* ^+/+^ and *Casp9* ^−/−^ MEFs were immunoblotted for pro‐caspase‐9 and ACTIN. (B) *Casp9* ^−/−^ MEFs were treated for the indicated times with brefeldin A (BFA), etoposide and paclitaxel or were γ‐irradiated and heat shocked followed by recovery for up to 72 h. Immunoblots for cleaved caspase‐8 and cleaved caspase‐3 are shown. *Casp9* ^−/−^ MEFs stably transduced with (C) empty vector (EV) and *Casp8* shRNA or with (D) EV and *Atg5* shRNA were treated with brefeldin A (BFA), etoposide and paclitaxel for the indicated times or γ‐irradiated and heat shocked followed by recovery for up to 72 h. Immunoblots for (C) cleaved caspase‐8, pro‐caspase‐8, cleaved caspase‐3 or (D) ATG5‐ATG12, cleaved caspase‐8 and caspase‐3 are shown. ACTIN was used as a loading control

We previously reported that prolonged ER stress induced by thapsigargin (Tg) or tunicamycin (Tm) leads to processing of pro‐caspase‐8 and pro‐caspase‐3 in *Casp9* ^−/−^ MEFs.^10^ Here, we investigated whether ER stress (BFA), DNA damage (etoposide and γ‐irradiation), microtubule stabilization (paclitaxel) and heat shock could similarly activate caspase‐8 in the absence of caspase‐9 (Figure 1A). We observed that all treatments increased the levels of the cleaved form of caspase‐8 and of caspase‐3 (Figure 1B) and induction of cell death, albeit with slower kinetics than for *Casp9* ^+/+^ MEFs (Figure S1).

We have also previously reported that ER stress‐induced caspase‐3 processing in *Casp9* ^−/−^ MEFs is dependent on caspase‐8 and ATG5.^10^ Here, shRNA‐mediated silencing of caspase‐8 in *Casp9* ^−/−^ MEFs resulted in a reduction in pro‐caspase‐3 processing in response to BFA, etoposide, paclitaxel or subjected cells to γ‐irradiation and heat shock (Figure 1C). Similarly, we knocked down ATG5 in *Casp9* ^−/−^ MEFs and treated cells with diverse stressors. shRNA‐mediated silencing of ATG5 produced a large reduction in the ATG5‐ATG12‐conjugated protein, the most abundant form of cellular ATG5^23^ (Figure 1D). In control cells (empty vector), stress stimuli led to an increase in the levels of ATG5‐ATG12 and also increased the processing of pro‐caspase‐8 (Figure 1D). By contrast, in sh*Atg5* MEFs, there was almost complete inhibition of pro‐caspase‐8 (and pro‐caspase‐3) processing, indicating a requirement for ATG5 in caspase‐8 and caspase‐3 activation under diverse stresses (Figure 1D).

### 3.2 Stressosome formation is a common event in *Casp9^−/−^* MEFs upon induction of cell death with diverse stimuli

**FIGURE 2:**
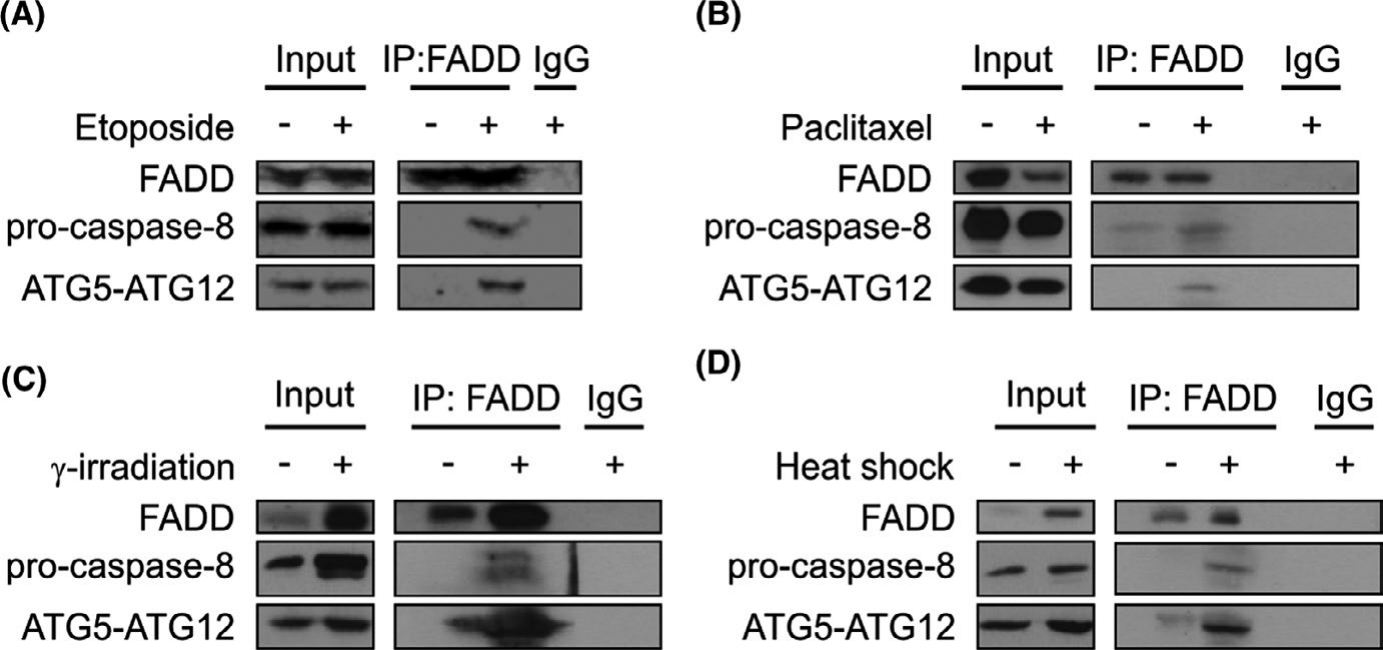
Stressosome formation in *Casp9* ^−/−^ MEFs in response to different stress stimuli. *Casp9* ^−/−^ MEFs were treated with vehicle, etoposide and paclitaxel for 72 h or γ‐irradiated and heat shocked followed by recovery for 72 h in the presence of Boc‐D‐FMK (20 μM, added 48 h before cell lysis). Proteins were immunoprecipitated with either control IgG or anti‐FADD antibodies. Immune complexes were analysed by immunoblotting for ATG5, FADD and pro‐caspase‐8.

We next assessed whether formation of the stressosome complex, containing pro‐caspase‐8, ATG5 and FADD,^10^ occurred with different stress stimuli. Immunoprecipitation of *Casp9* ^−/−^ MEF lysates with anti‐FADD (etoposide, paclitaxel, γ‐IR and heat shock) or anti‐ATG5 (BFA) antibodies revealed co‐precipitation of pro‐caspase‐8, ATG5‐ATG12 and FADD (Figure 2A‐D, Figure S2), indicating that stressosome formation is a common event in *Casp9* ^−/−^ MEFs irrespective of the initiating stress stimulus.

### 3.3 Computational modelling indicates how the stressosome components interact

**FIGURE 3:**
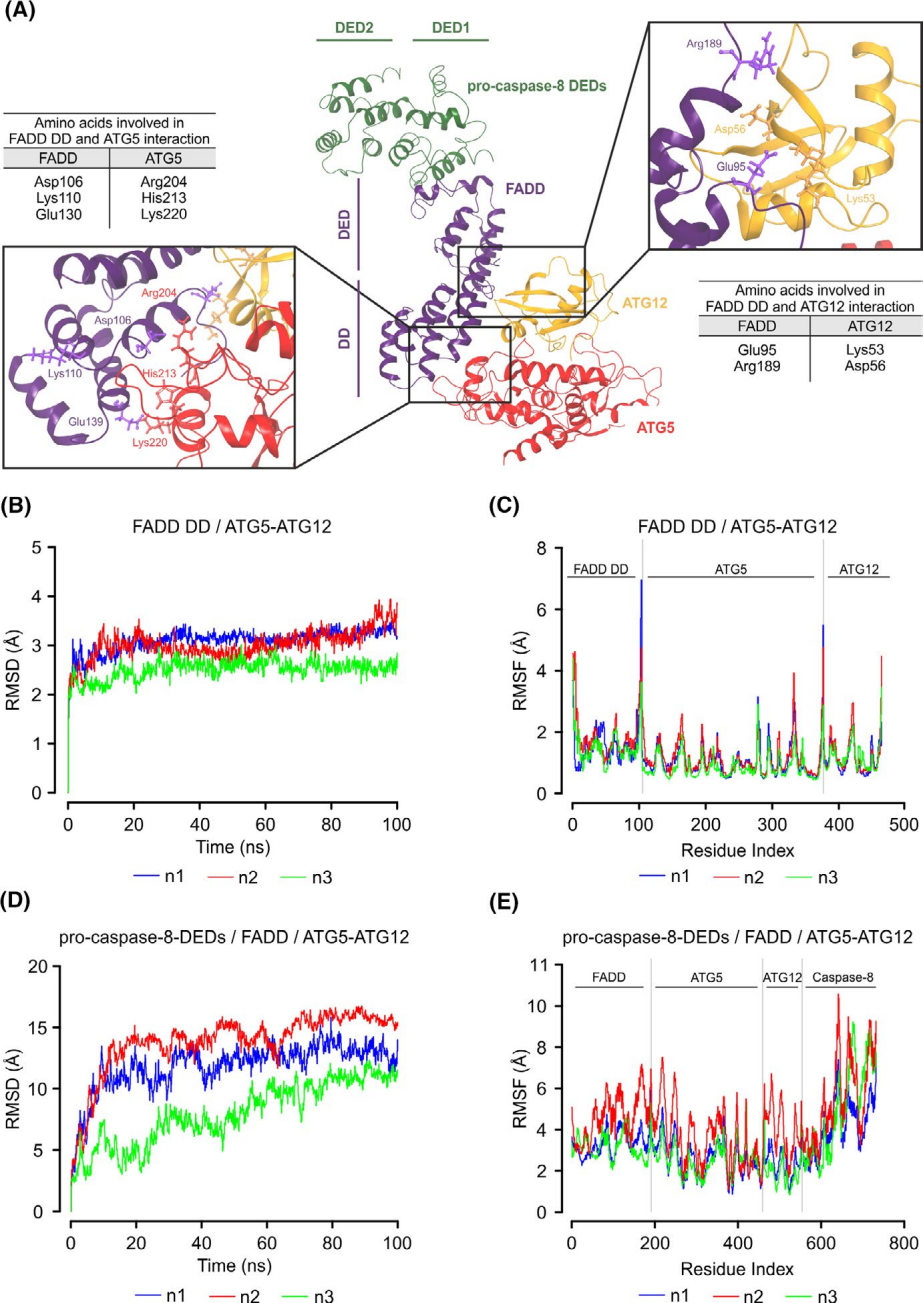
Molecular docking model of the core stressosome components. (A) Ribbon structure representing the complex of pro‐caspase‐8 death effector domains (DEDs) (PDB: 5L08) (green) and full‐length FADD (PDB: 2GF5) (violet) superposed with a complex of ATG5‐ATG12 (PDB: 4GDK) (red‐yellow) and FADD death domain (FADD DD) (PDB: 1E3Y). The key residues involved in the interaction between FADD and ATG5‐ATG12 are indicated (other residues are not labelled for clarity). (B) Root‐mean‐square deviation (RMSD) and (C) root‐mean‐square fluctuation (RMSF) of FADD DD/ATG5‐ATG12 complex. Data show three independent repeats. (D) RMSD and (E) RMSF of pro‐caspase‐8 DEDs/FADD/ATG5‐ATG12 complex. Data show three independent repeats

Protein docking studies were then used to identify the mode of interactions between stressosome components. We undertook a blind protein‐protein docking approach using PatchDock and FireDock webservers to predict the best docking complex between human ATG5‐ATG12 (PDB:4GDK) and FADD DD (PDB:1E3Y). The pose with the lowest docking score (−6.85 kcal/mol) was further validated by 100 ns MD simulation. Residue scanning predicts that the FADD DD binds to ATG5‐ATG12 through several points of interaction with both ATG5 and ATG12 components of the conjugated protein (Figure 3A). The main residues involved in the interaction are indicated in Figure 3A. Root‐mean‐square deviation (RMSD) and root‐mean‐square fluctuation (RMSF) results show that the complex remains stable (Figure 3B,C). Next, we identified the interaction pose between pro‐caspase‐8 DEDs (PDB:5L08) and full‐length FADD (PDB:2GF5). We performed a non‐blind protein‐protein docking using amino acids 2–13 and 40–51 in the pro‐caspase‐8 DED1 pocket and the F25/L26 motif in the DED domain of FADD, as described previously^18^ (Figure 3A). Further, we superposed the pro‐caspase‐8 DED/FADD complex with the previous FADD DD/ATG5‐ATG12 complex to obtain the merged stressosome core components (Figure 3A). MD simulations performed in triplicate show that the complex is stable. Despite the large fluctuations in RMSD, mostly due to flexibility between DED and DD domains of FADD and between DED1 and DED2 of pro‐caspase‐8, the RMSF results show that all proteins remain in interaction within the complex (Figure 3D,E).

### 3.4 Lack of dependence of caspase‐8 activation on autophagy or the integrated stress response

**FIGURE 4:**
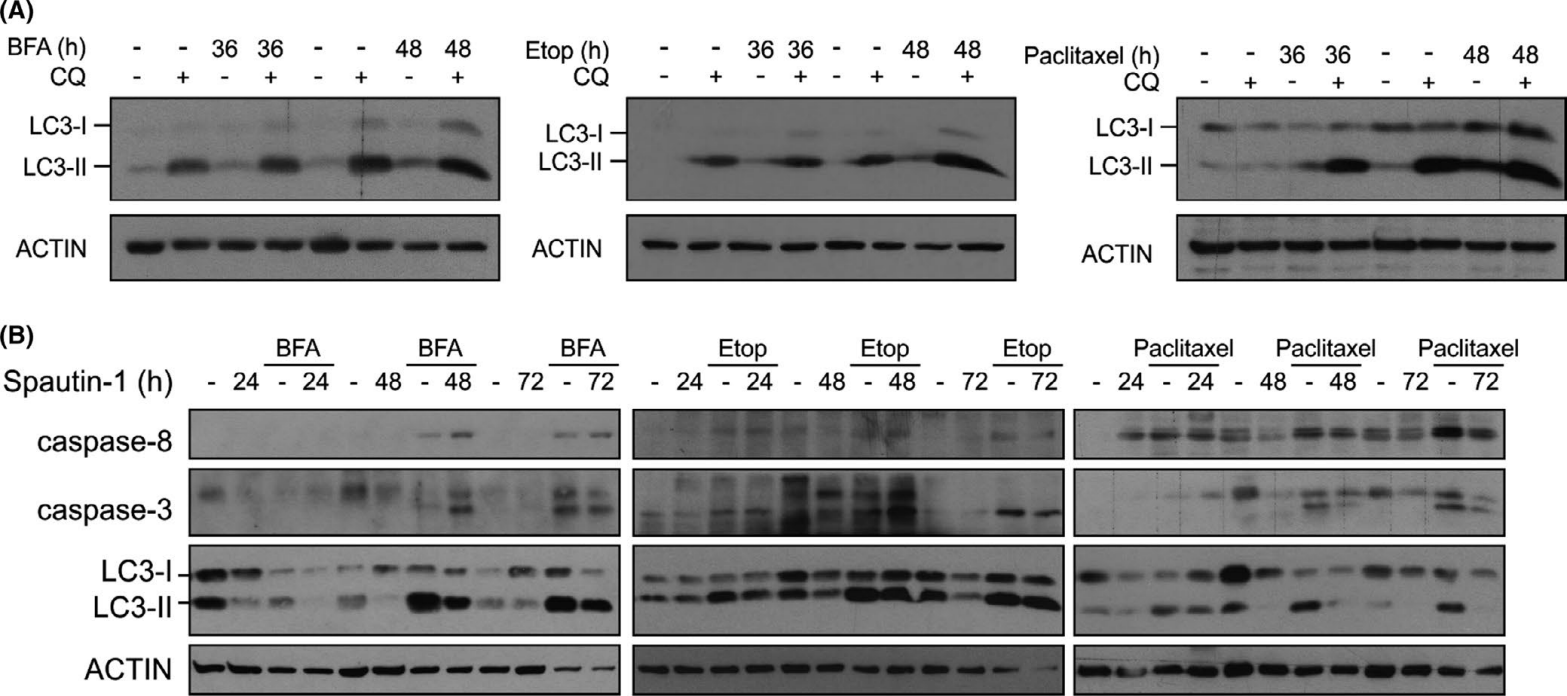
Inhibition of autophagy at early stages does not inhibit caspase‐8 activation. *Casp9* ^−/−^ MEFs were treated with brefeldin A (BFA), etoposide and paclitaxel alone or in a combination with (A) 20 μM chloroquine and (B) 10 μM Spautin‐1 for the indicated times. Whole cell lysates were immunoblotted for (A) light‐chain 3 (LC3)‐I to LC3‐II conversion, (B) cleaved caspase‐8 and caspase‐3 and LC3. ACTIN was used as loading control

We next investigated whether stressosome formation is dependent on autophagy. Treatment of *Casp9* ^−/−^ MEFs with BFA, etoposide or paclitaxel triggered autophagic flux as determined by conversion of LC3‐I to the lipidated LC3‐II form in the presence of the lysosomal inhibitor chloroquine (Figure 4A). Inhibition of ATG5‐ATG12 recruitment to the pre‐autophagosomal structures using Spautin‐1^24^ did not affect the kinetics or extent of caspase‐8 and caspase‐3 processing (Figure 4B), while it was effective in reducing LC3 conversion in response to BFA, etoposide and paclitaxel (Figure 4B) to the similar extent as knockdown of *Atg5* (Figure S3). These data suggest that while components of the autophagy pathway are involved in stressosome complex formation autophagy itself is not essential for caspase activation.

**FIGURE 5:**
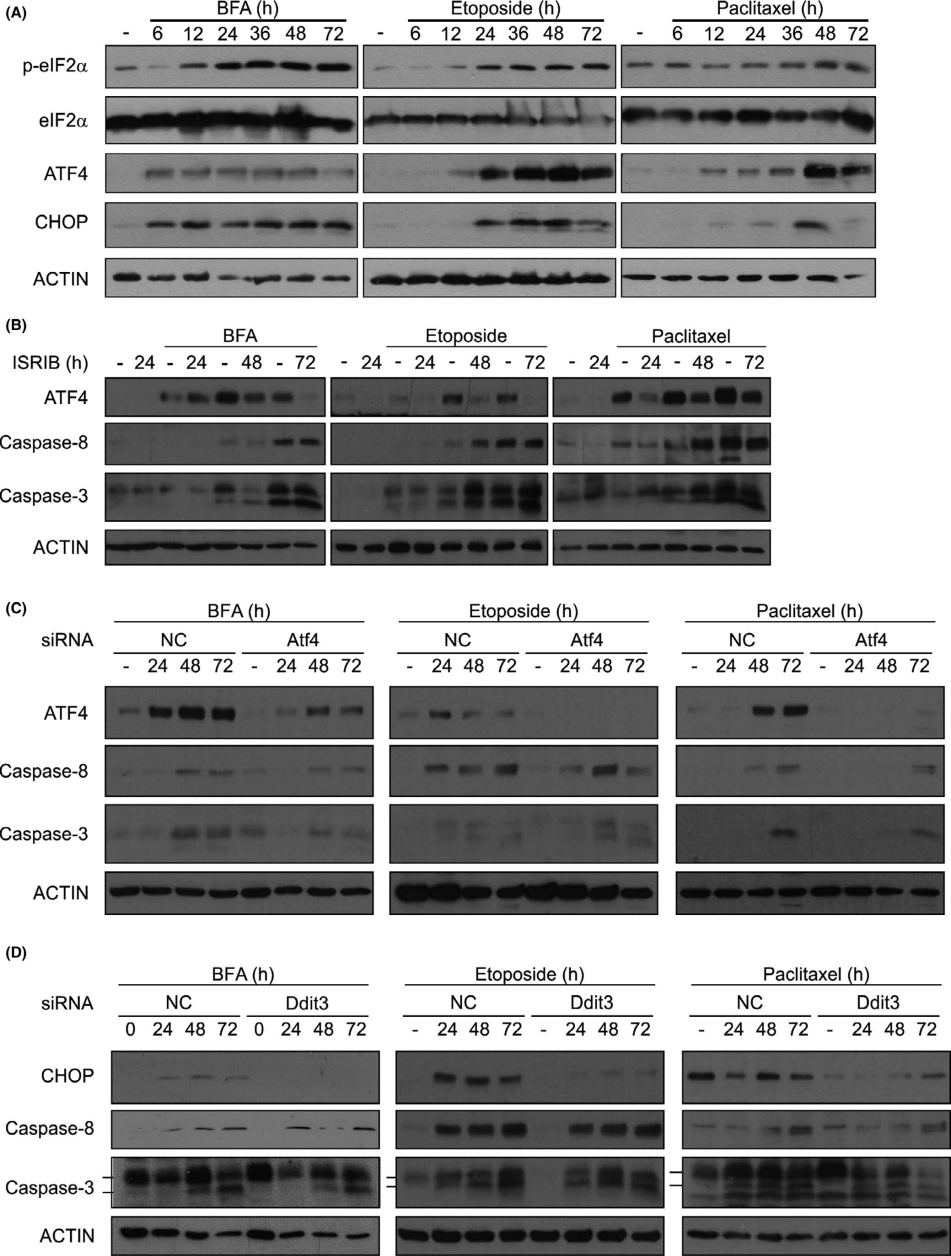
Inhibition of the ISR does not prevent caspase‐8 activation. (A) *Casp9* ^−/−^ MEFs were treated with brefeldin A (BFA), etoposide or paclitaxel for the indicated times. Whole cell lysates were immunoblotted for p‐eIF2α, eIF2α, ATF4, CHOP and ACTIN. (b‐d) *Casp9* ^−/−^ MEFs were treated with 200 nM integrated stress response inhibitor (ISRIB) (B) or transfected with non‐coding (NC) and *Atf4* siRNA (C) or *Ddit3* siRNA (D) followed by treatment with BFA, etoposide or paclitaxel for 24–72 h. Immunoblots show (B and C) ATF4, cleaved caspase‐8, cleaved caspase‐3 or (D) CHOP, cleaved caspase‐8 and caspase‐3. ACTIN was used as loading control

The ISR is activated by an array of diverse stress stimuli and can ultimately lead to caspase activation and cell death.^25^ Indeed, we observed that treatment of *Casp9* ^−/−^ MEFs with BFA, etoposide, paclitaxel and γ‐irradiation induced phosphorylation of eIF2α, expression of ATF4 and CHOP, the core components of ISR signalling (Figure 5A). To assess a potential role for the ISR in activation of caspase‐8, we used pharmacological and genetic approaches to inhibit ISR signalling. We found that inhibition of the ISR using ISRIB^26^ reduced expression of ATF4 but did not affect activation of caspase‐8 and −3 by BFA, etoposide or paclitaxel (Figure 5B). Knockdown of *Atf4* (Figure 5C) or *Ddit3* (Figure 5D) in *Casp9* ^−/−^ MEFs also did not affect processing of caspase‐8 and caspase‐3 induced by indicated stressors. Together our data suggest that the ISR does not contribute to the downstream activation of caspases.

### 3.5 Effect of caspase‐8 and ATG5 knockdown on cell death induction by various stressors

**FIGURE 6:**
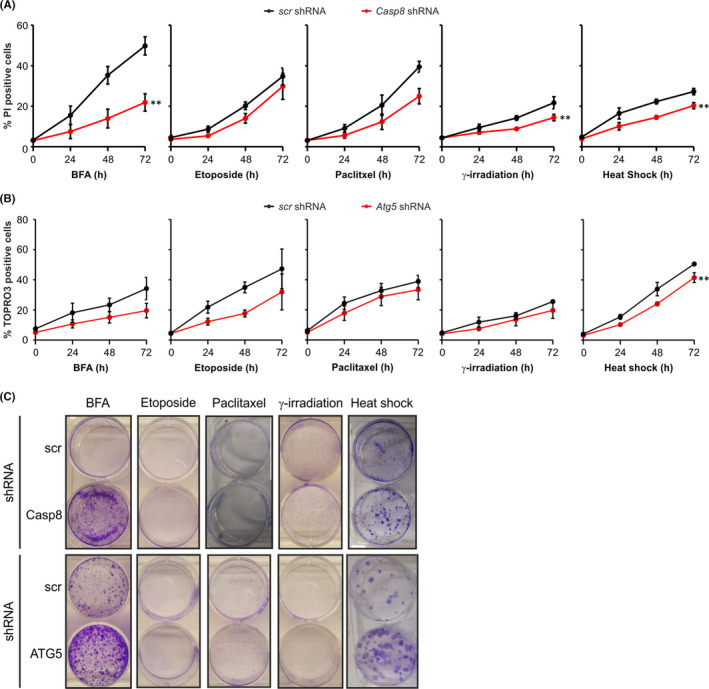
The role of the stressosome in cell death of *Casp9* ^−/−^ MEFs is dependent on the type of stress stimuli. *Casp9* ^−/−^ MEFs stably transduced with scrambled shRNA and (A, C) *Casp8* shRNA or (B, C) *Atg5* shRNA were treated with BFA, etoposide, paclitaxel for the indicated times or exposed to γ‐irradiation or heat shock and allowed to recover for up to 72 h. (A) PI or (B) ToPro3 uptake was analysed at the indicated time points after treatment. (C) Following treatment for 72 h the culture medium was changed and cells were left to form colonies. Clonogenic survival assay was performed 10 days later

Due to the role of caspases in apoptosis, we next examined whether stressosome‐dependent caspase‐8 activation mediates the cell death observed upon exposure of *Casp9* ^−/−^ MEFs to various stresses (Fig. S1). We knocked down caspase‐8 or ATG5 to analyse cell death via PI uptake and long‐term survival by looking at colony formation. While knockdown of *Casp8* significantly reduced cell death due to BFA, γ‐irradiation, heat shock and a small extent paclitaxel, it did not affect cell death induced by etoposide (Figure 6A). *Atg5* knockdown cells displayed reduced sensitivity to cell death induced by BFA, etoposide and heat shock but similar sensitivity to cell death induced by paclitaxel and γ‐irradiation as control counterparts (Figure 6B). We also observed that, upon depletion of caspase‐8 or ATG5, more colonies were formed following BFA and heat‐shock treatments (Figure 6C). A comparable number of *Casp8* or *Atg5* knockdown cells and control cells survived paclitaxel treatment and exposure to γ‐irradiation, but they did not form colonies due to the primary inhibitory effect of these treatments on proliferation (Figure 6C). Following treatment with etoposide, there was no long‐term survival of cells.

### 3.6 Stressosome‐mediated caspase‐8 activation is regulated by cFLIP_L_

**FIGURE 7:**
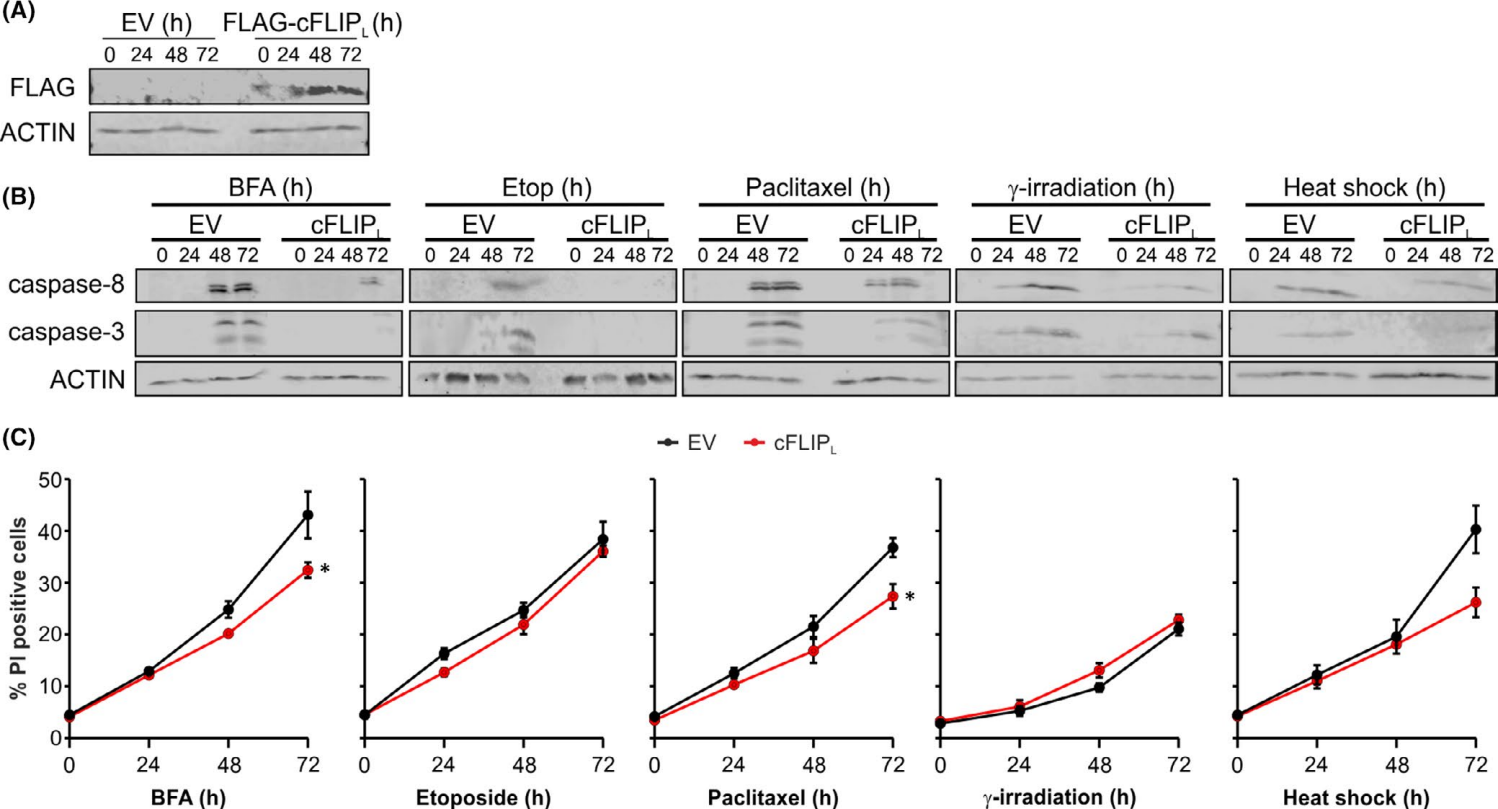
Overexpression of cFLIP_L_ leads to reduced stressosome‐mediated caspase activation. (A–C) *Casp9* ^−/−^ MEFs were transfected with empty vector (EV) or cFLIP_L_. (A) Whole cell lysates were immunoblotted for FLAG‐tag and ACTIN. (B and C) Following transfection *Casp9* ^−/−^ MEFs were treated with brefeldin A (BFA), etoposide and paclitaxel for the indicated times or exposed to γ‐irradiation or heat shock followed by incubation under standard conditions for up to 72 h. (B) Immunoblots show cleaved caspase‐8 and caspase‐3 and ACTIN. (C) Analysis of PI uptake was performed at the indicated time points after treatment

The molecular model (Figure 3A) indicated that cFLIP could prevent caspase‐8 activation within the stressosome complex by interacting with the α1/α4 helices of caspase‐8 DED2, thereby blocking filament formation or, alternatively, by direct binding to FADD DED instead of caspase‐8. To test this, we overexpressed FLAG‐tagged the long form of cFLIP (cFLIP_L_) in *Casp9* ^−/−^ MEFs (Figure 7A) and showed that cFLIP_L_‐overexpressing cells displayed reduced processing of pro‐caspase‐8 and pro‐caspase‐3 following treatment with BFA, etoposide, paclitaxel or exposure to γ‐irradiation or heat shock compared to empty vector counterparts (Figure 7B). cFLIP_L_ was also able to reduce cell death in response to BFA, paclitaxel and heat shock but not in response to etoposide or γ‐irradiation (Figure 7C).

## 4 DISCUSSION

Here, we demonstrate that diverse cellular stresses can lead to the formation of an alternative caspase‐8‐activating platform, which we have termed the stressosome. Formation of this complex is unmasked when the intrinsic apoptosis pathway is blocked.

Molecular docking supports a model of the stressosome in which pro‐caspase‐8 interacts with FADD through their respective DEDs, and in which the DD of FADD has several points of interaction with both ATG5 and ATG12. However, the model does not explain how multiple pro‐caspase‐8 molecules attain suitable close proximity in the stressosome for auto‐activation. One possibility is that the stressosome forms on autophagosomal membranes through an interaction between ATG5‐ATG12 and ATG16L. Based on the model, ATG5‐ATG12 bound to FADD would be physically available for interaction with dimeric ATG16L.^27^ This suggests that ATG5‐ATG12:ATG16L complex localized on pre‐autophagosomal structures might serve as a platform for stressosome assembly. In support of this, some groups have reported the involvement of pre‐autophagosomal membranes in ATG5‐mediated activation of caspase‐8.^3^, ^12^, ^13^ Young and colleagues showed that pro‐caspase‐8 forms a complex with ATG5 and FADD on ATG16L‐ and LC3‐positive autophagosomal membranes in cells proficient in apoptosis.^3^, ^9^ However, in our experiments, inhibition of autophagosome formation did not reduce pro‐caspase‐8 processing and reduced caspase‐8 activation was observed only in the absence of ATG5 (Figure 4). These findings indicate that the autophagic conjugation machinery but not autophagosomes are required for caspase‐8 activation and that ATG5‐mediated autophagosome elongation and stressosome formation occur independently of each other.

A number of reports suggest that caspase‐8 activation might require DR5 upregulation in response to ER stress,^14^, ^28^, ^29^, ^30^ but this may not be a universal feature of ER stress.^31^ The DR5 upregulation is driven by CHOP through the ISR signalling.^14^ In our experiments, we did not observe a role for the ISR in pro‐caspase‐8 processing.

One intriguing observation from our data is that while different stress stimuli lead to stressosome formation and caspase activation, cell death induction was not universally a consequence of stressosome formation. One explanation may be that stressosome‐dependent caspase activation in certain scenarios could initiate other downstream signalling pathways. It has been reported previously that caspase‐8 has additional, non‐apoptotic functions such as regulation of tumour cell motility, regulation of the inflammasome and cleavage of inflammatory interleukin‐1β.^32^, ^33^ Therefore, it is possible that formation of the stressosome and consequent activation of caspase‐8 could lead to inflammatory signalling rather than cell death, depending on the nature of the stimulus.

Resistance to apoptosis is a hallmark of cancer.^34^ Certain cancers express high levels of anti‐apoptotic proteins such as anti‐apoptotic Bcl‐2 family members or cFLIP or conversely exhibit loss of caspase‐9 and Apaf‐1.^35^, ^36^, ^37^, ^38^ Such cells frequently exhibit reduced or delayed cell death when exposed to chemotherapeutic drugs. Based on our findings, we suggest that stimulation of stressosome formation could circumvent the chemoresistance and make those cells more sensitive to treatment. For example, our data suggest that targeting cFLIP_L_ may be a strategy to sensitize cells to stressosome‐mediated cell death. cFLIP_L_ is frequently overexpressed in solid tumours and haematological cancers, and its high expression correlates with poor prognosis,^38^ and several approaches are under development to prevent caspase‐8 interaction with cFLIP_L_.^38^, ^39^

In conclusion, these findings highlight that cells resistant to intrinsic apoptosis can use alternative pathways to activate caspases. The stressosome complex, which consists of ATG5‐ATG12:FADD:pro‐caspase‐8, is formed in response to diverse stresses. Formation of the complex is independent of the ISR, while its activity can be blocked by cFLIP_L_. The full implications of this to cellular outcomes in cancer remain to be elucidated as caspase‐8 activation is involved in several cellular processes including cell death and inflammatory responses. However, having delineated the stressosome components and pathways regulating stressosome assembly may offer new targets to bypass chemoresistance in cancer.

## CONFLICT OF INTEREST

A.M.G., L.A.E. and A.S. are co‐founders and directors at Cell Stress Discoveries Ltd. They declare no conflict of interest.

## AUTHOR CONTRIBUTION

**Katarzyna Mnich:** Formal analysis (equal); Investigation (equal); Visualization (equal); Writing‐original draft (equal); Writing‐review & editing (equal). **Izabela Koryga:** Formal analysis (equal); Investigation (equal); Writing‐review & editing (equal). **Karolina Pakos‐Zebrucka:** Formal analysis (equal); Investigation (equal); Methodology (equal). **Melissa Thomas:** Formal analysis (equal); Investigation (equal); Methodology (equal); Writing‐review & editing (equal). **Susan Logue:** Formal analysis (equal); Funding acquisition (supporting); Investigation (equal); Writing‐review & editing (equal). **Leif Eriksson:** Conceptualization (equal); Supervision (equal); Writing‐review & editing (equal). **Adrienne M Gorman:** Conceptualization (equal); Funding acquisition (lead); Project administration (equal); Supervision (equal); Writing‐original draft (equal); Writing‐review & editing (equal). **Afshin Samali:** Conceptualization (lead); Funding acquisition (lead); Project administration (equal); Resources (equal); Supervision (equal); Writing‐review & editing (equal).

## Supporting information

Fig S1. *Casp9* ^+/+^ MEFs undergo delayed cell death induced by different stressors.*Casp9* ^+/+^ MEFs were treated with brefeldin A (BFA), etoposide, paclitaxel for the indicated times or γ‐irradiated and heat shocked followed by recovery for up to 72 h. Analysis of PI uptake was performed at the indicated time points after treatment.

Fig S2. Stressosome assembly in *Casp9* ^+/+^ MEFsin response to ER stress. *Casp9* ^+/+^ MEFs were treated with vehicle or brefeldin A (BFA) for up to 48 h in combination with Boc‐D‐FMK (20 μM, added 48 h before cell lysis). Proteins were immunoprecipitated with either control IgG or anti‐ATG5 antibodies. Immune complexes were analysed by immunoblotting for FADD, pro‐caspase‐8 and ATG5

Fig S3. Knockdown of ATG5 inhibits autophagy induced upon exposure of *Casp9* ^+/+^ MEFs to stress. *Casp9* ^+/+^ MEFs stably transduced with pGIPZ and *Atg5* shRNA were treated with BFA, etoposide, paclitaxel for 72 h alone or in a combination with 20 μM chloroquine. Whole cell lysates were immunoblotted for LC3‐I to LC3‐II conversion and ACTIN

## Data Availability

Data available on reasonable request from the authors.
