# Supplementary figures and images for: The stressosome, a caspase‐8‐activating signalling complex assembled in response to cell stress in an ATG5‐mediated manner
Source: J Cell Mol Med. 2021 Aug 7;25(18):8809–20. doi: 10.1111/jcmm.16840 (PMC8435408; doi:10.1111/jcmm.16840)

Mnich et al. Supplementary Figure 1

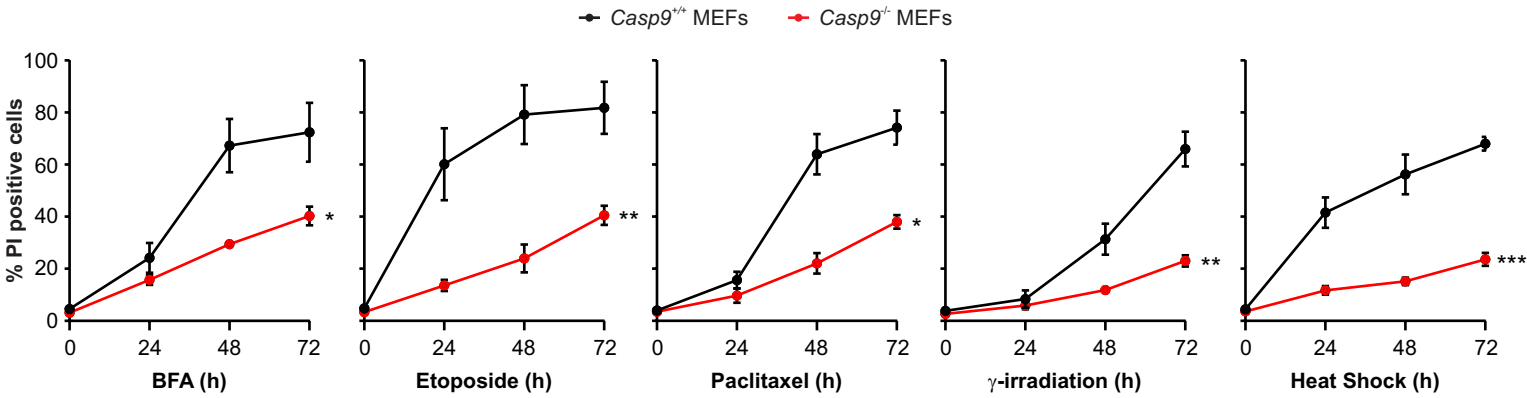

Supplement: Supplementary file 1 — Fig S1. Casp9 +/+ MEFs undergo delayed cell death induced by different stressors.Casp9 +/+ MEFs were treated with brefeldin A (BFA), etoposide, paclitaxel for the indicated times or γ‐irradiated and heat shocked followed by recovery for up to 72 h. Analysis of PI uptake was performed at the indicated time points after treatment. [file JCMM-25-8809-s003.pdf]

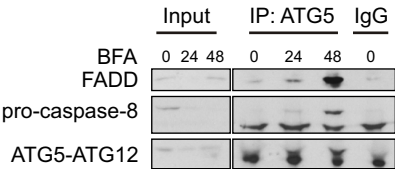

Supplement: Supplementary file 2 — Fig S2. Stressosome assembly in Casp9 +/+ MEFsin response to ER stress. Casp9 +/+ MEFs were treated with vehicle or brefeldin A (BFA) for up to 48 h in combination with Boc‐D‐FMK (20 μM, added 48 h before cell lysis). Proteins were immunoprecipitated with either control IgG or anti‐ATG5 antibodies. Immune complexes were analysed by immunoblotting for FADD, pro‐caspase‐8 and ATG5 [file JCMM-25-8809-s002.pdf]

Mnich et al. Supplementary Figure 3

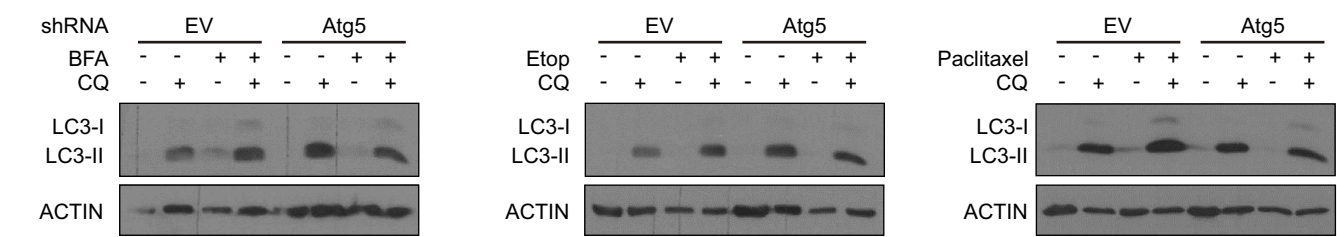

Supplement: Supplementary file 3 — Fig S3. Knockdown of ATG5 inhibits autophagy induced upon exposure of Casp9 +/+ MEFs to stress. Casp9 +/+ MEFs stably transduced with pGIPZ and Atg5 shRNA were treated with BFA, etoposide, paclitaxel for 72 h alone or in a combination with 20 μM chloroquine. Whole cell lysates were immunoblotted for LC3‐I to LC3‐II conversion and ACTIN [file JCMM-25-8809-s001.pdf]
